# Sociodemographic, psychological, and clinical characteristics associated with health service (non-)use for mental disorders in adolescents and young adults from the general population

**DOI:** 10.1007/s00787-023-02146-3

**Published:** 2023-02-18

**Authors:** H. Reich, H. C. M. Niermann, C. Voss, J. Venz, L. Pieper, K. Beesdo-Baum

**Affiliations:** 1 Institute of Clinical Psychology and Psychotherapy, Behavioral Epidemiology, Technische Universität Dresden, Chemnitzer Str. 46, 01187 Dresden, Germany; 2 Depression Research Centre of the German Depression Foundation, Department for Psychiatry, Psychosomatics and Psychotherapy, Goethe University, Heinrich-Hoffmann-Str. 10, Frankfurt am Main, 60528 Frankfurt, Germany

**Keywords:** Epidemiology, Mental health services, Adolescent, Young adult, Social stigma, Cross-sectional studies

## Abstract

Most adolescents and young adults who experience psychological distress do *not* seek professional help. This study aims to enhance the understanding of sociodemographic, psychological, and clinical characteristics associated with the underuse of health services by adolescents and young adults with mental disorders. Data from a cross-sectional, epidemiological study with a population-based sample (*N* = 1180 participants, 14–21 years old) were used. Participants completed a fully standardized, computer-assisted diagnostic interview (DIA-X-5/D-CIDI) administered by trained clinical interviewers to assess lifetime mental disorders according to DSM-5 as well as lifetime health service use for mental health problems, and completed self-report questionnaires to assess various psychological variables (e.g., stigma). Predictors of health service use were examined using univariate and multiple logistic regression analyses, data were weighted for age and sex to improve representativeness Of *n* = 597 participants with any lifetime mental disorder, 32.4% [95% CI 28.4; 36.7] had ever used any health services because of a mental health, psychosomatic, or substance use problem. Even less had received psychotherapeutic or pharmacological treatment (Cognitive Behavioral Therapy: 12.1% [9.5; 15.2]; other psychotherapy: 10.7% [8.4; 13.7]; medication: 5.4% [3.7; 7.8]). High education was associated with less health service use (low/ middle/ other vs. high education: 53.8% vs. 26.9%; OR = 0.26, *p* < .001). In the multiple regression model, stigma toward mental disorders was the single psychological variable associated with a reduced likelihood of using health services (OR = 0.69 [0.52; 0.90], *p* < .01). These findings draw attention to the treatment gap for mental disorders during adolescence and highlight related factors to be addressed in public health contexts.

**Supplementary Information:**

The online version contains supplementary material available at 10.1007/s00787-023-02146-3.

## Introduction

Most adolescents and young adults who experience psychological distress do *not* seek professional help [1]. Population-based surveys showed that only approximately one-third of adolescents with mental disorders received health services [2], and even in the presence of suicidal thoughts and self-harm less than half of affected individuals sought professional help [3]. Although half of all lifetime cases of mental disorders start by the age of 14 years [4], adolescents and young adults with a mental disorder were less likely to use services than older and middle aged adults [5]. Mental disorders during adolescence and young adulthood have been shown to represent risk factors for later psychiatric problems, drug use, suicide attempts, and premature termination of schooling [6, 7]. Therefore, treatment is urgently needed. Yet, negative attitudes toward the use of mental health services are especially prominent in adolescents and young adults [8]. Understanding the barriers to health service use on the individual level is crucial to reduce this mental health treatment gap.

Higher levels of psychological distress, a more severe impairment, and having more than one disorder predicted health service use in adolescents and young adults [1, 2, 9, 10]. Individuals with conduct disorders, oppositional/defiant disorders, or attention-deficit/hyperactivity disorders (ADHD) were more likely to seek professional treatment [2, 9], while service utilization was lower for anxiety, eating, or substance use disorders [2]. However, help-seeking behavior cannot be explained by severe emotional distress *alone*, as it has been shown to be influenced by sociodemographic and psychological variables [10].

Females were more likely than males to use services when mentally distressed [1]. Help-seeking behavior increased with age for females, while it decreased with age for males [11, 12]. Adolescents and young adults from ethnic minorities had a reduced likelihood to obtain mental health treatment compared to those from the ethnic majority of the population [13]. Service use for young persons with mental health needs was not found to be related to household income [14].

Not only sociodemographic, but also several psychological factors can function as barriers toward health service use for mental disorders in adolescents and young adults. Stigma and feelings of embarrassment were among the most important barriers toward help-seeking for mental disorders in young people [15]. *Personal stigma* (i.e., people’s own stigmatizing attitudes) was associated with a lower likelihood of using psychotropic medication, therapy, or counseling among college students [16]. *Perceived public stigma* (i.e., an individual’s perception of public stigma) was not associated with service use among college or university students though [16, 17]. High levels of personal control, a preference for self-reliance, and holding beliefs that one should be able to sort out their mental health problems on their own were further factors associated with low professional help-seeking for mental health problems in young people [15, 18, 19]. On the other hand, willingness to disclose and feeling emotionally competent to express their feelings [18], social support and encouragement from others [15], and beliefs that treatment can help have been shown to facilitate mental health service use in adolescents [19].

Most of the cited studies have used either convenience or specific clinical samples, and focused on only few possible predictors simultaneously in their analyses studying help-seeking behavior. The current study will assess a holistic range of sociodemographic, psychological, and clinical characteristics that are possibly associated with the underuse of health services in adolescents and young adults with mental disorders from the general population. Specifically, we will (1) report on health service use for mental disorders among adolescents and young adults from a community-based general population sample in Germany, and (2) examine sociodemographic, psychological, and clinical characteristics associated with health service (non-)use. Based on prior literature, we expect that only around one-third of adolescents and young adults with any mental disorder report mental health service use and that demographic factors (female sex, higher age), clinical characteristics (higher comorbidity, diagnosis of ADHD or any Disruptive, Impulse-Control or Conduct Disorder), and psychological factors (less stigma, lower internal and higher external control beliefs, higher emotional competence, and higher social support) are linked to service use.

## Methods

### Study population

Cross-sectional epidemiological data from *N* = 1,180 participants aged 14 to 21 years of the Behavior and Mind Health (BeMIND) study were used [20]. In 2015, an age- and sex-stratified random sample was drawn from the population registry of Dresden, Germany, and assessed between November 2015 and December 2016 (response/participation rate: 21.7%, cooperation rate: 42.8%). Participation was higher among females and those with higher education; lack of time and lack of interest were the most frequently given reasons for non-participation. For more details on sampling and recruitment procedures, please see Beesdo-Baum et al. [20].

### Procedures

In the study center at the Technische Universität Dresden, two assessment days were conducted approximately one week apart including a standardized diagnostic interview, self-report questionnaires, cognitive paradigms, and bio-sampling (blood and hair samples). Ecologic Momentary Assessment in everyday life and an online questionnaire assessment took place between these two in-person appointments. Participants received 50 Euro as incentive.

### Measures

An updated version of the fully standardized computer-assisted Munich-Composite International Diagnostic Interview (DIA-X/M-CIDI [21, 22]) was conducted face-to-face by trained clinical interviewers accompanied by tablet-based self-administered lists and questionnaires (DIA-X-5/D-CIDI [23]). Reliability and validity of the instrument have been established previously [22–24].

#### Lifetime mental disorders

The following diagnostic categories (including the respective mental disorders) were assessed according to DSM-5 criteria [25]: Substance Use Disorders (including tobacco use disorder, alcohol use disorder, any illegal substance use disorder), Possible Psychotic Disorder, Bipolar Disorders (bipolar I and II disorder), Depressive Disorders (major depressive disorder, persistent depressive disorder/ dysthymia), Anxiety Disorders (agoraphobia, social anxiety disorder, panic disorder, generalized anxiety disorder, specific phobias, other phobic anxiety disorder (situational type), separation anxiety disorder), Obsessive–Compulsive Disorder (OCD), Trauma-related disorders (acute stress disorder, post-traumatic stress disorder), Somatic Symptom or related disorders (somatic symptom disorder, illness anxiety disorder), Eating Disorders (anorexia nervosa, bulimia nervosa, binge eating disorder), Attention-Deficit Hyperactivity Disorder (ADHD; predominantly inattentive type, predominantly hyperactive type, mixed type), and Disruptive, Impulse-Control or Conduct Disorders (oppositional defiant disorder, intermittent explosive disorder, conduct disorder (childhood-onset and adolescent-onset type), antisocial personality disorder). For the present analyses, comorbidity was operationalized as having diagnoses in two or more diagnostic categories.

#### Health service use

Lifetime health service use for mental disorders was assessed in section Q of the DIA-X-5 interview by asking the following gate question while viewing a list of service institutions on the tablet screen: ‘Have you ever visited/contacted any of the health service institutions [as listed on the tablet screen] because of mental health, psychosomatic, or substance use problems, either by yourself, or through recommendation of others, e.g., medical doctors, relatives, or your partner?’. Health service use included inpatient treatment (e.g., psychiatric, neurological, psychotherapeutic, or psychosomatic hospitals), mental health outpatient treatment (e.g., delivered by psychiatric/psychotherapeutic outpatient clinics, resident psychiatrist/neurologist/psychotherapist/psychologist, and/or primary care physician), and complementary health services (e.g., different counseling centers). Individuals endorsing the gate question were further asked if they had been treated with cognitive-behavioral therapy (CBT), other psychotherapy, and/ or medication. Individuals who denied the gate question were asked if they had ever thought about using health services because of mental health, psychosomatic, or substance use problems.

#### Sociodemographic factors

The DIA-X-5/D-CIDI section A (see above) and self-report questionnaires delivered information on sociodemographic and psychological variables. Participants completed one part of the questionnaires on a tablet during on-site sessions and another part online between sessions. Education was dichotomized in high education (terminated or current education in higher secondary school, high school, or university) vs. low/middle/other education (terminated or current education in regular secondary school or complemented elementary school) for the means of the present analyses. The self-assessment of social class (lowest, lower middle, middle, upper middle, upper) was collapsed into three categories for the present analyses (low, middle, high) to achieve cell sizes no smaller than 5%. Migration background was categorized as present when one or both parents were not born in Germany [26].

#### Psychological correlates

The following psychological constructs were assessed using self-report questionnaires:

- Self-esteem: The Single-Item Self-Esteem Scale (SISE) is a one-item measure of global self-esteem [27] that was translated into German language for the present study. It was rated on a 7-point Likert scale (1 = ”not very true of me”, 7 = ”very true of me”). Retest reliability and construct validity have been established [27].
- Locus of control: Locus of control was assessed by the Internal–External Locus of Control-4 (IE-4) [28] with its’ two subscales Internal Control (Cronbach’s alpha in the current sample: alpha_BeMIND_ = 0.66) and External Control (alpha_BeMIND_ = 0.51). Each subscale had two items that were answered on a 5-point Likert scale (1 = ”strongly disagree”, 5 = ”fully agree”). The factor structure, reliability, and construct validity have been established by previous research [28].
- Emotion regulation: The Emotion-Regulation Skills Questionnaire (ERSQ; German version: [29]) was used. Its 27 items assess nine competencies relevant for a successful emotion regulation. These were answered on a 5-point Likert scale (0 = ”not at all”, 4 = ”(almost) always”, alpha_BeMIND_ = 0.94). The reliability and validity of the ERSQ have been confirmed [29–31].
- Social support: The seven item-version of the Social Support Questionnaire (F-SozU; [32]) was used to assess the subjectively perceived or anticipated social support from the social network. It was answered on a 5-point Likert scale (1 = ”strongly disagree”, 5 = ”fully agree”, alpha_BeMIND_ = 0.88). The factor structure, reliability, and construct validity of the F-SozU have been established [33].
- Stigma: Personal stigma associated with mental disorders was assessed with one item (“I would be ashamed if I had a mental disorder”) which was answered on a visual analog scale with labeled endpoints (0 = ”that’s not at all like me”, 10 = ”that’s very much like me”).
- Subjective physical health: One item (“How would you describe your physical health in general?”) which was answered on a 5-point Likert scale (1 = ”excellent”, 5 = ”bad”) was used to assess subjective physical health.

### Statistical analyses

Data were weighted to improve representativeness regarding sex and age (for details on weighting and representativeness of the sample see Beesdo-Baum et al. [20]). Only absolute frequencies are reported unweighted. Psychological scale variables were z-standardized. Predictors of health service use were examined using logistic regression analyses with weighted data. We report univariate analyses to inform about the effects of predictors by themselves and multiple analyses that included all variables in a single model to examine the contribution of each predictor when adjusted for all other, non-collinear variables as covariates. Odds ratios (ORs) and 95% confidence intervals [95% CI] are given.

All analyses were carried out for the whole sample and for both sexes separately as differences in health service use between sexes and within age groups have been reported before. The calculations of weighted row percentages (%w_row_) and logistic regression analyses were based on cases with complete information on the respective predictor variables (for sociodemographic and psychological variables, min. 11 and max. 78 individuals with any mental disorder did not provide data, for resulting sample sizes in the respective analyses, please refer to Table 2). Missing data for health service use were counted as no occurrence (twelve individuals with any mental disorder had not completed DIA-X-5/D-CIDI section Q on health service use). Significance was set at *α* = 0.05. As our epidemiological study is of exploratory nature, no multiplicity adjustment was applied [34]. All analyses were carried out with Stata version 15.1 [35].

## Results

### Sample characteristics

The BeMIND-study sample consisted of *N* = 1,180 adolescents and young adults aged 14 to 21 years (age_mean±SD_ = 17.9 ± 2.3 years), with *n* = 685 (48.3%) being female. The majority of participants had a high education (*n* = 882, 78.7%) and was living with their parents (*n* = 881, 65.1%), while *n* = 109 participants (12.5%) lived alone and *n* = 50 (5.4%) lived with a partner (other living arrangement: *n* = 140, 17.1%). *n* = 134 participants (11.5%) had a migration background. The perceived social class was low for *n* = 167 participants (17.2%), medium for *n* = 710 participants (60.6%), and high for *n* = 278 participants (22.2%).

### Health service use

The following data are presented in Table 1 (whole sample) and Tables S1/S2 (by sex). According to the standardized clinical-diagnostic interview, *n* = 303 participants had a lifetime diagnosis in one diagnostic category and *n* = 294 participants had lifetime diagnoses in two or more diagnostic categories. *n* = 583 participants reported no lifetime mental disorder. Of *n* = 597 participants with any lifetime mental disorder, *n* = 193 (32.4% [28.4; 36.7]) had ever used health services because of a mental health, psychosomatic, or substance use problem.

**Table 1:** Health service use because of mental health, psychosomatic, or substance use problems in participants with any mental disorder (*N* = 597)

	Total	Having ever used services				Having ever received psychological or medical treatment												Having ever thought about using services (without service utilization)			
						CBT				Other psychotherapy				Medication							
	*N*	*N*	%w_row_	[95% CI]		*N*	%w_row_	[95% CI]		*N*	%w_row_	[95% CI]		*N*	%w_row_	[95% CI]		*N*	%w_row_	[95% CI]	
Sociodemographics																					
Sex																					
Female	373	129	36.5	31.5	41.9	56	**16.1**	12.5	20.5	47	13.3	10.0	17.4	20	5.9	3.8	9.2	60	**16.5**	12.9	20.8
Male	224	64	28.4	22.5	35.2	16	**7.5**	4.4	12.3	19	7.8	4.8	12.3	10	4.7	2.4	8.8	18	**7.6**	4.7	12.1
Age																					
14–17 years	263	84	32.9	27.3	39.1	26	9.7	6.6	14.0	29	11.0	7.7	15.6	8	3.1	1.5	6.1	34	12.6	9.0	17.2
18–21 years	334	109	32.5	27.4	38.1	46	13.0	9.7	17.2	37	10.5	7.5	14.4	22	6.4	4.1	9.7	44	12.0	8.9	16.0
Education																					
Low/middle/other	134	65	**53.8**	44.3	63.1	28	**23.9**	16.5	33.2	17	13.9	8.5	21.9	12	10.1	5.5	17.6	9	5.8	2.9	11.1
High	441	122	**26.9**	22.7	31.5	43	**8.8**	6.5	11.9	46	9.6	7.1	12.9	18	4.2	2.6	6.7	66	13.9	10.9	17.6
Social class																					
Low	100	44	41.2	31.3	52.0	19	17.5	11.0	26.8	15	15.1	8.9	24.7	6	5.9	2.5	13.5	14	15.6	9.1	25.3
Middle	343	102	29.9	24.9	35.4	36	10.6	7.5	14.7	33	8.9	6.3	12.5	16	4.6	2.8	7.6	41	10.7	7.9	14.5
High	142	42	31.5	23.6	40.5	16	10.9	6.5	17.7	14	9.3	5.3	15.7	7	6.4	2.9	13.6	21	12.9	8.3	19.5
Migration background																					
No	509	166	32.9	28.6	37.5	60	11.8	9.1	15.1	54	10.3	7.8	13.4	28	5.7	3.8	8.3	61	10.9	8.4	14.0
Yes	88	27	30.9	21.1	42.8	12	13.0	7.0	22.9	12	12.7	6.8	22.4	2	3.1	0.7	13.5	17	20.1	12.3	31.1
Psychopathology																					
Diagnostic category																					
Any substance use disorder	266	87	31.2	25.6	37.4	33	11.1	7.9	15.5	32	11.2	7.8	15.7	14	5.5	3.2	9.5	34	11.2	7.9	15.7
Psychotic disorder	67	24	34.5	22.9	48.2	11	17.8	9.5	30.8	7	11.8	5.2	24.7	5	7.1	2.8	17.0	12	16.0	8.8	27.4
Any bipolar disorder	18	8	50.4	25.2	75.3	3	18.6	5.2	48.8	2	12.0	2.4	42.9	1	7.8	0.9	44.3	4	17.5	5.5	43.7
Any depressive disorder	190	83	46.2	38.5	54.0	34	19.1	13.6	26.2	34	18.2	12.9	24.9	17	9.2	5.6	14.8	32	16.7	11.7	23.1
Any anxiety disorder	279	105	39.4	33.4	45.9	49	18.1	13.7	23.6	32	11.4	8.0	16.2	19	7.7	4.8	12.2	45	15.8	11.8	20.9
OCD	66	31	49.0	36.0	62.2	18	26.3	16.5	39.1	10	15.2	7.9	27.3	8	12.1	5.8	23.5	8	12.1	5.9	23.1
Any trauma- or stressor-related disorder	50	25	53.2	38.2	67.7	15	35.3	21.9	51.4	11	25.3	13.8	41.8	2	5.3	1.3	19.5	14	23.0	13.3	36.8
Any somatic symptom or related disorder	59	27	52.8	38.5	66.7	15	30.4	18.5	45.5	12	21.2	11.8	35.1	4	7.3	2.5	19.9	10	15.0	7.8	26.8
Any eating disorder	33	24	73.4	54.1	86.6	15	47.6	29.8	66.0	10	28.5	14.9	47.7	5	16.2	6.4	35.2	4	13.0	4.5	32.3
ADHD	16	10	58.0	28.0	83.1	2	8.2	1.6	32.6	4	31.1	10.3	64.0	5	33.8	12.1	65.4	3	11.3	2.9	35.2
Any disruptive. impulse-control or conduct disorder	110	49	41.6	32.1	51.7	17	14.4	8.8	22.7	16	13.3	7.9	21.4	8	8.4	4.1	16.5	11	10.1	5.5	18.0
Number of diagnostic categories																					
One	303	65	22.2	17.4	27.8	22	6.7	4.3	10.4	18	5.5	3.4	8.9	6	1.9	0.8	4.5	27	8.2	5.5	12.0
Two	152	55	35.0	27.2	43.6	13	8.1	4.5	14.1	23	13.6	9.0	20.1	9	5.9	3.0	11.3	23	14.4	9.5	21.3
Three or more	142	73	51.9	42.9	60.8	37	26.6	19.4	35.3	25	18.2	12.1	26.3	15	11.8	7.0	19.4	28	18.2	12.5	25.8

*%w*_*row*_ weighted row percentages. *OCD* obsessive–compulsive disorder. *ADHD* attention-deficit hyperactivity disorder. *CBT* cognitive behavioral therapy. To facilitate readability, proportions for which 95% CIs are not overlapping are printed in bold in the sociodemographic section

#### CBT or other psychotherapeutic treatment

Of *n* = 597 participants with any lifetime mental disorder, *n* = 72 participants (12.1% [9.5; 15.2]) reported to have received CBT and *n* = 66 (10.7% [8.4, 13.7]) to have been treated with other psychotherapy. The proportions for receiving CBT were higher among females than males, and higher among participants with low/middle/other education than among participants with high education.

#### Treatment with medication

Only *n* = 30 participants (5.4% [3.7; 7.8]) with any lifetime mental disorder reported pharmacological treatment.

#### Intentions to use health services

Of those with any lifetime mental disorder, 12.3% [9.8; 15.3] had been *thinking about* using health services because of mental health, psychosomatic, or substance use problems without ever doing it. The proportion of females thinking about using health services was 16.5% [12.9; 20.8], while it was 7.6% [4.7; 12.1] in males.

### Factors associated with health service use

Sociodemographic, clinical, and psychological factors were analyzed regarding their association with health service use because of mental health, psychosomatic, or substance use problems in all participants with a mental disorder (see Table 2), as well as for female (see Table S3) and male participants with a mental disorder (see Table S4).

**Table 2:** Factors associated with health service use because of mental health, psychosomatic, or substance use problems in participants with any mental disorder

	Univariate (unadjusted)					Multiple (adjusted for covariates)				
	*N*	OR	[95% CI]		*p*	*N*	OR	[95% CI]		*p*
Sociodemographics										
Sex (male vs. female)	585	0.69	0.47	1.01	0.059	466	0.92	0.54	1.58	0.774
Age (14–21)	585	1.02	0.94	1.11	0.608	466	1.05	0.93	1.18	0.453
Education (high vs. low/middle/other)	564	**0.32**	**0.20**	**0.49**	**< 0.001**	466	**0.26**	**0.15**	**0.47**	** < 0.001**
Social class (low = ref.)	574	1.00				466	1.00			
(Middle)	574	**0.61**	**0.37**	**1.00**	**0.049**	466	0.88	0.45	1.70	0.696
(High)	574	0.65	0.37	1.17	0.151	466	1.12	0.51	2.46	0.779
Migration background (yes vs. no)	585	0.91	0.53	1.57	0.737	466	0.81	0.39	1.67	0.569
Psychopathology (lifetime)										
Any substance use disorder	585	0.88	0.61	1.28	0.507	466	1.00	0.60	1.67	0.993
Psychotic disorder	585	1.10	0.61	1.98	0.759	466	0.53	0.23	1.22	0.136
Any mood disorder^a^	585	**2.49**	**1.69**	**3.67**	** < 0.001**	466	**1.94**	**1.15**	**3.27**	**0.013**
*Any bipolar disorder*	585	2.15	0.78	5.93	0.140					
*Any depressive disorder*	585	**2.35**	**1.59**	**3.48**	** < 0.001**					
Any anxiety disorder	585	**1.74**	**1.19**	**2.53**	**0.004**	466	1.35	0.83	2.22	0.226
OCD	585	**2.17**	**1.24**	**3.81**	**0.007**	466	1.65	0.74	3.69	0.224
Any trauma- or stressor-related disorder	585	**2.56**	**1.37**	**4.79**	**0.003**	466	1.79	0.75	4.27	0.191
Any somatic symptom or related disorder	585	**2.56**	**1.40**	**4.66**	**0.002**	466	1.52	0.62	3.74	0.357
Any eating disorder	585	**6.27**	**2.73**	**14.39**	** < 0.001**	466	**5.13**	**1.90**	**13.85**	**0.001**
ADHD or any disruptive, impulse-control or conduct disorder^a^	585	**1.67**	**1.07**	**2.60**	**0.024**	466	**1.89**	**1.05**	**3.42**	**0.035**
*ADHD*	585	**2.96**	**0.94**	**9.34**	**0.064**					
*Any disruptive, impulse-control or conduct disorder*	585	**1.65**	**1.04**	**2.60**	**0.033**					
Number of diagnostic categories^b^ (one = ref.)	585	**1.00**								
(Two)	585	**1.89**	**1.18**	**3.02**	**0.008**					
(Three or more)	585	**3.80**	**2.38**	**6.05**	** < 0.001**					
Psychological variables and physical health										
Global self-esteem (SISE)	513	**0.73**	**0.61**	**0.88**	**0.001**	466	0.96	0.75	1.24	0.765
Internal locus of control (IE-4)	511	**0.74**	**0.62**	**0.90**	**0.002**	466	0.93	0.71	1.23	0.614
External locus of control (IE-4)	511	**1.43**	**1.18**	**1.74**	** < 0.001**	466	1.09	0.85	1.41	0.488
Emotion regulation (SEK-27)	507	**0.72**	**0.59**	**0.89**	**0.002**	466	0.90	0.67	1.20	0.459
Social support (F-SOZU)	507	**0.74**	**0.61**	**0.91**	**0.003**	466	0.84	0.64	1.09	0.194
Stigma (“I’d be ashamed if I had a mental disorder”)	555	**0.78**	**0.63**	**0.96**	**0.019**	466	**0.69**	**0.52**	**0.90**	**0.007**
Subjective physical health	516	**1.37**	**1.11**	**1.68**	**0.003**	466	1.18	0.90	1.55	0.221

*OR* odds ratio. [95% CI]: 95% confidence interval. *OCD* obsessive–compulsive disorder. *ADHD* attention-deficit hyperactivity disorder. Significant results are printed in bold

^a^Diagnostic categories that correspond to ICD-10 F3-disorders (Any Bipolar Disorder, Any Depressive Disorder) and F9-disorders (ADHD, Any Disruptive, Impulse-Control or Conduct Disorder) enter as only one category each into multiple regression analyses due to small cell sizes of Any Bipolar Disorder and ADHD; see Table 1)

^b^Number of diagnostic categories doesn’t enter into multiple regression analyses due to collinearity with diagnostic categories

^c^All scales are standardized

#### Sociodemographic factors

On a descriptive level, males were less likely than females to use health services (28.4 vs. 36.5%). Age was unrelated to health service use report in the whole sample, but sex-specific univariate analyses showed that health service use was higher in older than younger females (31.0 vs. 39.4%), while the opposite was observed for males (35.3 vs. 25.5%). High education and perceived middle or high social class were associated with less health service use. The effects of social class were especially prominent in females. No differences in health service use due to migration background were observed in the present data.

#### Psychopathology

Participants with mental disorders in more than one diagnostic category were more likely to use health services than participants with diagnoses in only one diagnostic category (one category: 22.2%; two categories: 35.0%, OR_unadj._ = 1.89, *p* = 0.008; three or more categories: 51.9%, OR_unadj._ = 3.80, *p* < 0.001). The effect of comorbid diagnostic categories on health service use was especially prominent in females. After adjusting for covariates, the diagnostic categories any Mood Disorder (OR_adj._ = 1.94, *p* = 0.013), any Eating Disorder (OR_adj._ = 5.13, *p* = 0.001), and ADHD or any Disruptive, Impulse-Control or Conduct Disorder (OR_adj._ = 1.89, *p* = 0.035) were related to more health service use. The only single diagnostic category that was associated with a *reduced* likelihood for using health services was any Substance Use Disorder in males; this effect was no longer significant when adjusting for covariates.

#### Psychological variables

After adjusting for covariates, stigma was the single psychological variable that was associated with a reduced likelihood of using health services (OR_adj._ = 0.69, *p* = 0.007). In univariate analyses, all psychological variables were significantly associated with health service use in the whole sample. However, the pattern of statistically significant effects varied between sexes: Health service use in females was associated with low self-esteem, lowered emotion regulation skills, external control beliefs, and bad subjective physical health. In males, social support and internal control beliefs were associated with a reduced likelihood to use health services.

## Discussion

As expected, only one-third of adolescents and young adults with any lifetime mental disorder had ever used health services because of mental health, psychosomatic, or substance use problems in the present study [2, 9]. Our contribution adds that even fewer participants received a treatment recommended according to the guidelines for most mental disorders, with 1–2 out of 10 participants with a mental disorder receiving psychotherapy, and 1 out of 20 participants with a mental disorder receiving pharmacological treatment. Albeit effective treatment options for most mental disorders exist and can be used as a service of public health insurance free of charge in Germany, health service use was low. Possible reasons for the limited health service use rates in these general population samples of youth might include lower severity, impairment, or persistence in many cases with a mental disorder; yet at least a diagnostic evaluation by a health care professional would be desirable given the known long-term outcomes of mental disorders during this crucial developmental period [6].

Key sociodemographic and psychological factors associated with low health service use despite fulfilling the DSM-5 criteria for a mental disorder were high education and high personal stigma. Stigma associated with mental disorders has been widely recognized as one of the main psychological barriers in taking the decision to seek help for mental health problems [36]. Other psychological variables were associated with health service use in univariate analyses only, mirroring results of previous studies using smaller sample sizes and thus a smaller set of explanatory variables. In the current study, self-esteem, control beliefs, emotion regulation, and social support were no longer statistically significant correlates of help-seeking behavior when adjusting for psychopathology according to DSM-5. Mood disorders in particular are highly prevalent and often co-occurring with other disorder categories. Depressive disorders are inherently characterized by changes in, e.g., self-esteem [37] and emotion regulation [31]. When DSM-5 psychopathology was included in multiple analyses, single psychological variables did not contribute unique shares of variance to explain health service use anymore. Noteworthy, stigma represented an independent construct and played a crucial role regarding the explanation of health service use, independently of disorder categories and sociodemographic characteristics. Recent findings from the UK showed that socioeconomic disadvantage was linked to higher service use in young people [10]. Our results were able to replicate this in a representative sample from the general population. Possible explanations for higher health service use include higher exposure to psychosocial (violence, family turmoil, child separation from family) and physical (noise, crowding, sub-standard housing) risk-factors during adolescence in socioeconomically disadvantaged youth, which may lead to more mental health problems [38]. Another hypothesis is that more sources of non-professional help might be available to highly educated participants, which may be associated with less professional help-seeking.

In line with previous studies, we did not find a statistically significant effect for sex [39, 40], but less health service use was observable on a descriptive level in the male subsample, especially for any Substance Use Disorder. Social norms and traditional masculinity are possible barriers to help-seeking [41], and previous research has indicated few health service use among male adolescents for DSM-IV alcohol abuse/dependence [2]. Particularly for boys, recognizing their emotional state and having a vocabulary to explain it, seems to be a problem and a barrier toward help-seeking [42]. As we expected, an increase of health service use with age was found in females only. This might be explained by a faster development of females in puberty and them reaching a phase of more autonomy earlier [11]. Concurring with previous research, comorbidity and diagnosis of ADHD or any Disruptive, Impulse-Control or Conduct Disorder were linked with higher service use [1, 2, 9, 10]. Health service use for any Eating Disorder was higher (73% for all services, 48% CBT-treatment) in the current sample than reported in the United States (13%, [2]). A diagnosis of any Mood Disorder was also liked with higher service use in the present sample.

The current study provides a holistic perspective on health service use as it presents analyses of data from a general population sample of adolescents and young adults and not only at-risk groups or clinical samples. There are some limitations to address though. Further psychological variables that might be associated with health service use (e.g., mental health literacy) have not been assessed. Personal stigma was assessed with one item only. Many psychological variables are closely related to diagnostic categories (e.g., self-esteem and depression), which might be a reason why they were not significant predictors of health service use in multiple analyses. The presented data are of cross-sectional nature and future analyses need to assess the predictive value of the associated factors to establish their risk factor status in longitudinal designs. For a discussion of the representativeness of the BeMIND-sample, please refer to the BeMIND methods paper [20]. Briefly, females and those with higher education were more likely to participate in the study. Sex but not education was considered in sample weights. As high education was found to be related to less service use, service use rates might be somewhat underestimated in this current study. This, however, does not necessarily affect the validity of associations between high education and service non-use. This might concur with the hypothesis that individuals with higher education seek for other forms of contact in dealing with mental health difficulties (e.g., participating in a longitudinal study on mental health, using peer support, online self-help, and other forms of non-professional help-seeking) that have not been assessed in the present study. Apart from risk factors on the individual level, further barriers toward health service use exist on the societal and health system level. These were beyond the scope of the present study.

Our analyses contribute to the existing body of literature documenting the treatment gap for mental disorders during adolescence, a crucial transition period into adulthood. The lack of health service use might reflect a delay of help-seeking, i.e., a treatment *lag*, which may be due to a delay in the decision of adolescents and parents to seek help [12]. Low mental health literacy is closely related to stigmatizing attitudes [43] and need to be addressed urgently. So far, interventions to promote help-seeking are not effective among children and adolescents in terms of improving attitudes, intentions, and behaviors to seek professional help for mental health problems [44]. It is therefore critical to tailor anti-stigma and mental health literacy interventions to the needs of youth, who are actively calling for action themselves [45]. Mental health needs to be addressed by family doctors and pediatricians as well as in schools and in the training of teachers, who represent possible gatekeepers. For individuals at risk for mental illness, community-based collaborative care should address the needs of adolescents and young adults in a participatory and systemic approach.

### Supplementary Information

Below is the link to the electronic supplementary material.

Supplementary file1 (PDF 979 KB)

## Data Availability

The datasets analyzed during the current study are available from the corresponding author on reasonable request.
