# Supplementary material for: Sociodemographic, psychological, and clinical characteristics associated with health service (non-)use for mental disorders in adolescents and young adults from the general population
Source: Eur Child Adolesc Psychiatry. 2023 Feb 18;33(2):391–400. doi: 10.1007/s00787-023-02146-3 (PMC10869368; doi:10.1007/s00787-023-02146-3)
Supplement: Supplementary file 1 — Supplementary file1 (PDF 979 KB) [file 787_2023_2146_MOESM1_ESM.pdf]

*Supplementary material: Health service use and factors associated with health service use, shown separately for female and male participants.*

Article title: Sociodemographic, psychological, and clinical characteristics associated with health service (non-)use for mental disorders in adolescents and young adults from the general population

Journal Name: European Child & Adolescent Psychiatry

Authors: Reich, H., Niermann, H.C.M., Voss, C., Venz, J., Pieper, L., Beesdo-Baum, K.

Corresponding author: Prof. Dr. Katja Beesdo-Baum, Technische Universität Dresden, Institute of Clinical Psychology and Psychotherapy, Behavioral Epidemiology, Dresden, Germany. E-mail: katja.beesdo-baum@tu-dresden.de

Content:

Table S1. Health service use in females because of mental health, psychosomatic, or substance use problems (*N*=373 with a mental disorder). Page 2

Table S2. Health service use in males because of mental health, psychosomatic, or substance use problems (N=224 with a mental disorder). Page 3

Table S3. Factors associated with health service use among females because of mental health, psychosomatic, or substance use problems. Page 4

Table S4. Factors associated with health service use among males because of mental health, psychosomatic, or substance use problems. Page 5

*Table S1. Health service use in females because of mental health, psychosomatic, or substance use problems (N=373 with a mental disorder).*

| | Total | Having ever used services | | | | Having ever received psychological or medical treatment: CBT | | | | Having ever received psychological or medical treatment: Other psychotherapy | | | | Having ever received psychological or medical treatment: Medication | | | | Having ever thought about using services (without service utilization) | | | |
|---|---|---|---|---|---|---|---|---|---|---|---|---|---|---|---|---|---|---|---|---|---|
| | *N* | *N* | $\%w_{row}$ | [95% CI] | | *N* | $\%w_{row}$ | [95% CI] | | *N* | $\%w_{row}$ | [95% CI] | | *N* | $\%w_{row}$ | [95% CI] | | *N* | $\%w_{row}$ | [95% CI] | |
| **Sociodemographics** | | | | | | | | | | | | | | | | | | | | | |
| Age | | | | | | | | | | | | | | | | | | | | | |
| 14-17 years | 167 | 51 | 31.0 | 24.3 | 38.6 | 18 | 11.2 | 7.1 | 17.2 | 17 | 9.9 | 6.2 | 15.5 | 4 | 2.5 | 0.9 | 6.5 | 27 | 16.8 | 11.7 | 23.5 |
| 18-21 years | 206 | 78 | 39.4 | 32.7 | 46.5 | 38 | 18.7 | 13.7 | 24.8 | 30 | 15.0 | 10.6 | 20.9 | 16 | 7.7 | 4.7 | 12.5 | 33 | 16.3 | 11.7 | 22.2 |
| Education | | | | | | | | | | | | | | | | | | | | | |
| low/ middle/ other | 83 | 41 | **57.5** | 45.9 | 68.4 | 19 | 27.4 | 17.9 | 39.5 | 11 | 16.6 | 9.1 | 28.2 | 8 | 11.8 | 5.8 | 22.6 | 7 | 8.3 | 3.8 | 17.0 |
| high | 274 | 85 | **31.7** | 26.2 | 37.8 | 36 | 13.4 | 9.7 | 18.2 | 33 | 12.1 | 8.6 | 16.7 | 12 | 4.6 | 2.6 | 8.1 | 50 | 18.5 | 14.2 | 23.9 |
| Social class | | | | | | | | | | | | | | | | | | | | | |
| low | 58 | 30 | 52.1 | 38.3 | 65.5 | 16 | 28.3 | 17.5 | 42.4 | 11 | 19.1 | 10.4 | 32.5 | 4 | 6.4 | 2.2 | 17.1 | 8 | 15.1 | 7.4 | 28.2 |
| middle | 214 | 69 | 33.7 | 27.3 | 40.8 | 28 | 13.8 | 9.6 | 19.5 | 22 | 11.0 | 7.2 | 16.4 | 13 | 6.9 | 3.9 | 11.7 | 34 | 16.7 | 12.0 | 22.8 |
| high | 94 | 27 | 30.6 | 21.5 | 41.4 | 12 | 13.6 | 7.7 | 23.0 | 12 | 12.9 | 7.3 | 21.8 | 3 | 3.7 | 1.1 | 11.6 | 16 | 16.2 | 9.9 | 25.5 |
| Migration background | | | | | | | | | | | | | | | | | | | | | |
| no | 318 | 109 | 36.7 | 31.2 | 42.5 | 47 | 15.9 | 12.1 | 20.8 | 38 | 12.8 | 9.4 | 17.3 | 19 | 6.6 | 4.2 | 10.3 | 49 | 15.6 | 11.9 | 20.3 |
| yes | 55 | 20 | 35.8 | 23.4 | 50.4 | 9 | 17.1 | 8.7 | 31.1 | 9 | 16.2 | 8.1 | 29.9 | 1 | 1.7 | 0.2 | 11.8 | 11 | 21.5 | 11.8 | 36.1 |
| **Psychopathology** | | | | | | | | | | | | | | | | | | | | | |
| Diagnostic category | | | | | | | | | | | | | | | | | | | | | |
| Any Substance Use Disorder | 152 | 60 | 42.7 | 34.5 | 51.3 | 28 | 20.4 | 14.3 | 28.3 | 23 | 16.1 | 10.7 | 23.4 | 10 | 6.8 | 3.6 | 12.5 | 27 | 17.8 | 12.3 | 25.1 |
| Psychotic Disorder | 40 | 16 | 39.0 | 24.2 | 56.2 | 8 | 23.8 | 11.8 | 42.0 | 3 | 8.3 | 2.3 | 25.4 | 4 | 11.3 | 3.9 | 28.3 | 10 | 26.2 | 13.9 | 43.9 |
| Any Bipolar Disorder | 10 | 5 | 55.9 | 20.7 | 86.0 | 3 | 39.6 | 10.7 | 78.1 | 2 | 25.6 | 4.6 | 71.0 | 1 | 16.5 | 1.6 | 71.2 | 4 | 37.2 | 10.5 | 74.9 |
| Any Depressive Disorder | 144 | 66 | 48.8 | 40.1 | 57.5 | 28 | 20.3 | 14.2 | 28.3 | 29 | 21.9 | 15.4 | 30.1 | 12 | 8.8 | 4.9 | 15.2 | 26 | 18.7 | 12.8 | 26.4 |
| Any Anxiety Disorder | 201 | 80 | 41.9 | 34.8 | 49.3 | 41 | 22.1 | 16.5 | 28.9 | 27 | 13.5 | 9.2 | 19.3 | 14 | 7.3 | 4.3 | 12.2 | 38 | 19.5 | 14.4 | 26.0 |
| OCD | 45 | 19 | 46.4 | 31.3 | 62.1 | 14 | 35.4 | 21.7 | 52.0 | 7 | 16.1 | 7.4 | 31.5 | 5 | 10.5 | 4.2 | 23.9 | 5 | 11.6 | 4.6 | 26.3 |
| Any Trauma- or Stressor-related disorder | 40 | 22 | 58.8 | 42.0 | 73.8 | 13 | 37.0 | 22.3 | 54.6 | 8 | 20.9 | 10.1 | 38.3 | 2 | 7.2 | 1.7 | 25.5 | 11 | 23.6 | 12.6 | 39.7 |
| Any Somatic Symptom or related disorder | 45 | 20 | 49.5 | 33.7 | 65.3 | 11 | 27.4 | 15.3 | 44.2 | 10 | 23.6 | 12.6 | 39.7 | 2 | 3.6 | 0.8 | 14.2 | 9 | 20.2 | 10.3 | 35.8 |
| Any Eating Disorder | 33 | 24 | 73.4 | 54.1 | 86.6 | 15 | 47.6 | 29.8 | 66.0 | 10 | 28.5 | 14.9 | 47.7 | 5 | 16.2 | 6.4 | 35.2 | 4 | 13.0 | 4.5 | 32.3 |
| ADHD | 8 | 5 | 61.6 | 19.1 | 91.6 | 1 | 11.6 | 0.9 | 66.5 | 2 | 29.6 | 4.8 | 77.8 | 2 | 23.2 | 3.6 | 70.9 | 2 | 20.4 | 3.0 | 67.6 |
| Any Disruptive. Impulse-Control or Conduct Disorder | 49 | 28 | 58.2 | 43.1 | 72.0 | 12 | 26.5 | 15.2 | 42.1 | 10 | 22.2 | 11.8 | 37.7 | 3 | 8.4 | 2.6 | 23.6 | 6 | 13.8 | 6.0 | 28.6 |
| Number of diagnostic categories | | | | | | | | | | | | | | | | | | | | | |
| One | 173 | 33 | 19.4 | 13.9 | 26.5 | 15 | 8.5 | 5.1 | 13.9 | 10 | 6.2 | 3.3 | 11.5 | 2 | 0.9 | 0.2 | 3.7 | 18 | 10.3 | 6.4 | 16.0 |
| Two | 100 | 40 | 42.2 | 32.2 | 52.8 | 11 | 11.0 | 6.1 | 19.3 | 17 | 17.9 | 11.1 | 27.4 | 8 | 9.7 | 4.8 | 18.7 | 19 | 20.0 | 12.8 | 29.7 |
| Three or more | 100 | 56 | 58.5 | 48.0 | 68.3 | 30 | 32.8 | 23.6 | 43.5 | 20 | 20.2 | 13.1 | 30.0 | 10 | 10.3 | 5.4 | 18.7 | 23 | 23.0 | 15.4 | 32.9 |

*Note*. $\%w_{row}$: weighted row percentages. OCD: Obsessive Compulsive Disorder. ADHD: Attention Deficit Hyperactivity Disorder. CBT: Cognitive behavioural therapy. To facilitate readability, proportions for which 95%CIs are not overlapping are printed in bold in the sociodemographic section.

*Table S2. Health service use in males because of mental health, psychosomatic, or substance use problems (N=224 with a mental disorder).*

| | Total | Having ever used services | | | | Having ever received psychological or medical treatment | | | | | | | | | | | | Having ever thought about using services (without service utilization) | | | |
|---|---|---|---|---|---|---|---|---|---|---|---|---|---|---|---|---|---|---|---|---|---|
| | | | | | | CBT | | | | Other psychotherapy | | | | Medication | | | | | | | |
| | *N* | *N* | $\%w_{row}$ | [95% CI] | | *N* | $\%w_{row}$ | [95% CI] | | *N* | $\%w_{row}$ | [95% CI] | | *N* | $\%w_{row}$ | [95% CI] | | *N* | $\%w_{row}$ | [95% CI] | |
| **Sociodemographics** | | | | | | | | | | | | | | | | | | | | | |
| Age | | | | | | | | | | | | | | | | | | | | | |
| 14-17 years | 96 | 33 | 35.3 | 26.1 | 45.8 | 8 | 7.9 | 3.9 | 15.3 | 12 | 12.4 | 7.0 | 20.9 | 4 | 3.8 | 1.4 | 10.0 | 7 | 7.5 | 3.5 | 15.1 |
| 18-21 years | 128 | 31 | 25.5 | 18.3 | 34.4 | 8 | 7.3 | 3.6 | 14.2 | 7 | 5.8 | 2.7 | 12.1 | 6 | 5.0 | 2.2 | 10.9 | 11 | 7.7 | 4.2 | 13.8 |
| Education | | | | | | | | | | | | | | | | | | | | | |
| low/ middle/ other | 51 | 24 | **50.1** | 35.1 | 65.0 | 9 | **20.2** | 10.2 | 36.2 | 6 | 11.1 | 4.6 | 24.5 | 4 | 8.3 | 2.8 | 22.0 | 2 | 3.3 | 0.8 | 12.7 |
| high | 167 | 37 | **21.8** | 15.8 | 29.2 | 7 | **4.0** | 1.8 | 8.8 | 13 | 7.0 | 3.9 | 12.2 | 6 | 3.7 | 1.6 | 8.5 | 16 | 9.1 | 5.5 | 14.7 |
| Social class | | | | | | | | | | | | | | | | | | | | | |
| low | 42 | 14 | 31.5 | 18.4 | 48.4 | 3 | 7.8 | 2.3 | 23.4 | 4 | 11.6 | 4.2 | 28.4 | 2 | 5.5 | 1.3 | 20.6 | 6 | 16.0 | 7.1 | 32.4 |
| middle | 129 | 33 | 25.8 | 18.4 | 34.8 | 8 | 7.2 | 3.4 | 14.4 | 11 | 6.7 | 3.6 | 12.2 | 3 | 2.2 | 0.6 | 7.4 | 7 | 4.4 | 2.0 | 9.3 |
| high | 48 | 15 | 32.6 | 19.7 | 48.8 | 4 | 7.5 | 2.5 | 20.6 | 2 | 4.8 | 1.0 | 19.5 | 4 | 9.8 | 3.4 | 25.2 | 5 | 8.7 | 3.4 | 20.1 |
| Migration background | | | | | | | | | | | | | | | | | | | | | |
| no | 191 | 57 | 28.9 | 22.5 | 36.2 | 13 | 7.3 | 4.1 | 12.6 | 16 | 7.6 | 4.5 | 12.5 | 9 | 4.7 | 2.3 | 9.1 | 12 | 5.8 | 3.2 | 10.3 |
| yes | 33 | 7 | 25.7 | 11.8 | 47.2 | 3 | 8.7 | 2.2 | 28.3 | 3 | 9.0 | 2.5 | 27.7 | 1 | 4.7 | 0.6 | 28.8 | 6 | 18.6 | 7.9 | 37.9 |
| **Psychopathology** | | | | | | | | | | | | | | | | | | | | | |
| Diagnostic category | | | | | | | | | | | | | | | | | | | | | |
| Any Substance Use Disorder | 114 | 27 | 21.7 | 14.8 | 30.6 | 5 | 3.3 | 1.3 | 8.3 | 9 | 7.0 | 3.5 | 13.7 | 4 | 4.5 | 1.7 | 11.5 | 7 | 5.8 | 2.6 | 12.1 |
| Psychotic Disorder | 27 | 8 | 30.2 | 14.1 | 53.3 | 3 | 12.1 | 3.2 | 36.3 | 4 | 15.1 | 4.9 | 38.4 | 1 | 3.2 | 0.4 | 22.3 | 2 | 6.5 | 1.4 | 24.7 |
| Any Bipolar Disorder | 8 | 3 | 45.5 | 10.7 | 85.3 | 5 | 54.5 | 14.7 | 89.3 | 5 | 54.5 | 14.7 | 89.3 | 5 | 54.5 | 14.7 | 89.3 | 3 | 45.5 | 10.7 | 85.3 |
| Any Depressive Disorder | 46 | 17 | 41.1 | 26.5 | 57.5 | 6 | 16.8 | 7.5 | 33.6 | 5 | 11.0 | 4.2 | 25.8 | 5 | 10.1 | 3.9 | 23.8 | 6 | 12.8 | 5.4 | 27.3 |
| Any Anxiety Disorder | 78 | 25 | 35.2 | 24.4 | 47.8 | 8 | 11.4 | 5.4 | 22.3 | 5 | 7.9 | 3.2 | 18.3 | 5 | 8.5 | 3.5 | 19.3 | 7 | 9.6 | 4.4 | 19.6 |
| OCD | 21 | 12 | 52.8 | 29.0 | 75.5 | 4 | 12.7 | 4.1 | 33.3 | 3 | 13.9 | 3.8 | 40.1 | 3 | 14.4 | 3.9 | 40.7 | 3 | 12.8 | 3.7 | 36.1 |
| Any Trauma- or Stressor-related disorder | 10 | 3 | 37.6 | 9.7 | 77.2 | 2 | 30.6 | 6.1 | 74.8 | 7 | 62.4 | 22.8 | 90.3 | 7 | 62.4 | 22.8 | 90.3 | 3 | 21.4 | 4.8 | 59.3 |
| Any Somatic Symptom or related disorder | 14 | 7 | 59.8 | 28.1 | 85.0 | 4 | 36.3 | 12.5 | 69.4 | 2 | 16.4 | 3.2 | 54.2 | 2 | 14.9 | 2.8 | 51.3 | 1 | 4.4 | 0.4 | 32.1 |
| Any Eating Disorder | 0 | | | | | | | | | | | | | | | | | | | | |
| ADHD | 8 | 5 | 56.1 | 15.0 | 90.2 | 1 | 6.3 | 0.4 | 50.8 | 2 | 32.0 | 5.3 | 79.7 | 3 | 39.7 | 8.4 | 82.5 | 1 | 6.3 | 0.4 | 50.8 |
| Any Disruptive. Impulse-Control or Conduct Disorder | 61 | 21 | 32.7 | 21.4 | 46.4 | 5 | 7.8 | 3.0 | 18.9 | 6 | 8.5 | 3.5 | 19.1 | 5 | 8.5 | 3.3 | 20.0 | 5 | 8.1 | 3.2 | 19.1 |
| Number of diagnostic categories | | | | | | | | | | | | | | | | | | | | | |
| One | 130 | 32 | 24.5 | 17.4 | 33.4 | 7 | 5.2 | 2.3 | 11.3 | 8 | 4.9 | 2.3 | 10.2 | 4 | 2.7 | 0.9 | 7.6 | 9 | 6.4 | 3.2 | 12.3 |
| Two | 52 | 15 | 26.3 | 15.5 | 41.0 | 2 | 4.5 | 1.0 | 18.6 | 6 | 8.4 | 3.6 | 18.5 | 1 | 1.3 | 0.2 | 9.3 | 4 | 7.7 | 2.7 | 20.1 |
| Three or more | 42 | 17 | 41.9 | 26.7 | 58.9 | 7 | 17.3 | 7.8 | 34.2 | 5 | 15.0 | 6.1 | 32.5 | 5 | 14.1 | 5.7 | 30.9 | 5 | 11.0 | 4.2 | 25.7 |

*Note*. $\%w_{row}$: weighted row percentages. OCD: Obsessive Compulsive Disorder. ADHD: Attention Deficit Hyperactivity Disorder. CBT: Cognitive behavioural therapy. To facilitate readability, proportions for which 95%CIs are not overlapping are printed in bold in the sociodemographic section.

*Table S3. Factors associated with health service use among females because of mental health, psychosomatic, or substance use problems.*

| | **Univariate (unadjusted)** | | | | | **Multiple (adjusted for covariates)** | | | | |
|---|---|---|---|---|---|---|---|---|---|---|
| | *N* | OR | [95% CI] | | *p* | *N* | OR | [95% CI] | | *p* |
| **Sociodemographics** | | | | | | | | | | |
| Age (14 – 21) | 365 | **1.11** | **1.00** | **1.24** | **.044** | 299 | 1.04 | 0.88 | 1.22 | .662 |
| Education (high vs. low/ middle/ other) | 350 | **0.34** | **0.20** | **0.58** | **<.001** | 299 | **0.39** | **0.20** | **0.76** | **.006** |
| Social class (low=ref.) | 359 | 1.00 | | | | 299 | 1.00 | | | |
| (middle) | 359 | **0.47** | **0.25** | **0.87** | **.017** | 299 | 0.63 | 0.28 | 1.44 | .276 |
| (high) | 359 | **0.41** | **0.20** | **0.83** | **.014** | 299 | 0.51 | 0.19 | 1.39 | .186 |
| Migration background (yes vs. no) | 365 | 0.96 | 0.51 | 1.81 | .905 | 299 | 0.71 | 0.24 | 2.10 | .533 |
| **Psychopathology (lifetime)** | | | | | | | | | | |
| Any Substance Use Disorder | 365 | **1.60** | **1.01** | **2.52** | **.044** | 299 | 1.33 | 0.71 | 2.48 | .374 |
| Psychotic Disorder | 365 | 1.12 | 0.55 | 2.28 | .746 | 299 | 0.34 | 0.10 | 1.19 | .092 |
| Any Mood Disorder[a] | 365 | **2.54** | **1.60** | **4.03** | **<.001** | 299 | **1.86** | **1.00** | **3.45** | **.049** |
| *Any Bipolar Disorder* | 365 | 2.25 | 0.60 | 8.47 | .229 | | | | | |
| *Any Depressive Disorder* | 365 | **2.35** | **1.48** | **3.74** | **<.001** | | | | | |
| Any Anxiety Disorder | 365 | **1.66** | **1.05** | **2.63** | **.030** | 299 | 1.37 | 0.75 | 2.52 | .305 |
| OCD | 365 | 1.59 | 0.82 | 3.09 | .169 | 299 | 0.89 | 0.29 | 2.76 | .845 |
| Any Trauma- or Stressor-related disorder | 365 | **2.83** | **1.41** | **5.70** | **.004** | 299 | 2.71 | 1.00 | 7.35 | .050 |
| Any Somatic Symptom or related disorder | 365 | 1.84 | 0.94 | 3.62 | .075 | 299 | 1.33 | 0.51 | 3.46 | .556 |
| Any Eating Disorder | 365 | **5.66** | **2.43** | **13.18** | **<.001** | 299 | **6.22** | **2.14** | **18.08** | **.001** |
| ADHD or any Disruptive, Impulse-Control or Conduct Disorder[a] | 365 | **2.77** | **1.50** | **5.11** | **.001** | 299 | **3.84** | **1.68** | **8.80** | **.002** |
| *ADHD* | 365 | 2.85 | 0.63 | 12.92 | .174 | | | | | |
| *Any Disruptive, Impulse-Control or Conduct Disorder* | 365 | **2.85** | **1.50** | **5.42** | **.001** | | | | | |
| Number of diagnostic categories[b] (one=ref.) | 365 | 1.00 | | | | | | | | |
| (two) | 365 | **3.03** | **1.69** | **5.41** | **<.001** | | | | | |
| (three or more) | 365 | **5.85** | **3.29** | **10.41** | **<.001** | | | | | |
| **Psychological variables and physical health[c]** | | | | | | | | | | |
| Global self-esteem (SISE) | 328 | **0.74** | **0.60** | **0.92** | **.007** | 299 | 0.92 | 0.68 | 1.26 | .620 |
| Internal Locus of Control (IE-4) | 327 | 0.85 | 0.67 | 1.07 | .154 | 299 | 1.17 | 0.82 | 1.67 | .390 |
| External Locus of Control (IE-4) | 327 | **1.62** | **1.27** | **2.06** | **<.001** | 299 | 1.35 | 0.97 | 1.88 | .076 |
| Emotion regulation (SEK-27) | 325 | **0.68** | **0.54** | **0.87** | **.002** | 299 | 0.81 | 0.58 | 1.14 | .228 |
| Social support (F-SOZU) | 325 | 0.82 | 0.65 | 1.03 | .090 | 299 | 1.11 | 0.77 | 1.60 | .574 |
| Stigma ("I'd be ashamed if I had a mental disorder") | 347 | 0.84 | 0.67 | 1.05 | .132 | 299 | **0.71** | **0.52** | **0.96** | **.025** |
| Subjective physical health | 331 | **1.36** | **1.07** | **1.74** | **.013** | 299 | 1.21 | 0.89 | 1.63 | .224 |

*Note*. OR: Odds Ratio. [95% CI]: 95% Confidence Interval. OCD: Obsessive Compulsive Disorder. ADHD: Attention Deficit Hyperactivity Disorder. Significant results are printed in bold.

[a] Diagnostic categories that correspond to ICD-10 F3-disorders (Any Bipolar Disorder, Any Depressive Disorder) and F9-disorders (ADHD, Any Disruptive, Impulse-Control or Conduct Disorder) enter as only one category each into multiple regression analyses due to small cell sizes of Any Bipolar Disorder and ADHD; see Table 1). [b] Number of diagnostic categories doesn't enter into multiple regression analyses due to collinearity with diagnostic categories. [c] All scales are standardized.

*Table S4. Factors associated with health service use among males because of mental health, psychosomatic, or substance use problems.*

| | Univariate (unadjusted) | | | | | Multiple (adjusted for covariates) | | | | |
|---|---|---|---|---|---|---|---|---|---|---|
| | *N* | OR | [95% CI] | | *p* | *N* | OR | [95% CI] | | *p* |
| **Sociodemographics** | | | | | | | | | | |
| Age (14 – 21) | 220 | 0.93 | 0.82 | 1.06 | .286 | 167 | 1.00 | 0.81 | 1.24 | .964 |
| Education (high vs. low/ middle/ other) | 214 | **0.28** | **0.14** | **0.57** | **.001** | 167 | **0.16** | **0.05** | **0.46** | **.001** |
| Social class (low=ref.) | 215 | 1.00 | | | | 167 | 1.00 | | | |
| (middle) | 215 | 0.76 | 0.34 | 1.70 | .495 | 167 | 1.03 | 0.29 | 3.63 | .962 |
| (high) | 215 | 1.05 | 0.41 | 2.73 | .915 | 167 | 2.13 | 0.46 | 9.76 | .330 |
| Migration background (yes vs. no) | 220 | 0.85 | 0.32 | 2.23 | .743 | 167 | 0.83 | 0.26 | 2.60 | .743 |
| **Psychopathology (lifetime)** | | | | | | | | | | |
| Any Substance Use Disorder | 220 | **0.47** | **0.25** | **0.89** | **.020** | 167 | 0.85 | 0.34 | 2.14 | .729 |
| Psychotic Disorder | 220 | 1.10 | 0.42 | 2.90 | .847 | 167 | 0.75 | 0.21 | 2.68 | .652 |
| Any Mood Disorder[a] | 220 | **2.18** | **1.07** | **4.44** | **.032** | 167 | 2.82 | 0.97 | 8.20 | .057 |
| *Any Bipolar Disorder* | 220 | 2.16 | 0.46 | 10.14 | .330 | | | | | |
| *Any Depressive Disorder* | 220 | **2.10** | **1.00** | **4.37** | **.049** | | | | | |
| Any Anxiety Disorder | 220 | 1.62 | 0.85 | 3.11 | .143 | 167 | 1.19 | 0.47 | 3.05 | .710 |
| OCD | 220 | **3.18** | **1.18** | **8.60** | **.023** | 167 | 2.04 | 0.51 | 8.14 | .308 |
| Any Trauma- or Stressor-related disorder | 220 | 1.55 | 0.36 | 6.71 | .558 | 167 | 0.93 | 0.12 | 7.40 | .942 |
| Any Somatic Symptom or related disorder | 220 | **4.19** | **1.25** | **14.07** | **.021** | 167 | 1.64 | 0.30 | 9.08 | .568 |
| Any Eating Disorder[d] | 220 | - | | | | | | | | |
| ADHD or any Disruptive, Impulse-Control or Conduct Disorder[a] | 220 | 1.35 | 0.69 | 2.64 | .373 | 167 | 1.48 | 0.60 | 3.66 | .389 |
| *ADHD* | 220 | 3.40 | 0.70 | 16.51 | .129 | | | | | |
| *Any Disruptive, Impulse-Control or Conduct Disorder* | 220 | 1.33 | 0.67 | 2.63 | .410 | | | | | |
| Number of diagnostic categories[b] (one=ref.) | 220 | 1.00 | | | | | | | | |
| (two) | 220 | 1.10 | 0.51 | 2.39 | .810 | | | | | |
| (three or more) | 220 | **2.23** | **1.01** | **4.90** | **.047** | | | | | |
| **Psychological variables and physical health[c]** | | | | | | | | | | |
| Global self-esteem (SISE) | 185 | 0.77 | 0.55 | 1.08 | .132 | 167 | 1.00 | 0.64 | 1.55 | .994 |
| Internal Locus of Control (IE-4) | 184 | **0.64** | **0.47** | **0.88** | **.007** | 167 | 0.78 | 0.46 | 1.32 | .351 |
| External Locus of Control (IE-4) | 184 | 1.27 | 0.94 | 1.72 | .120 | 167 | 0.93 | 0.61 | 1.42 | .733 |
| Emotion regulation (SEK-27) | 182 | 0.80 | 0.56 | 1.14 | .212 | 167 | 1.04 | 0.61 | 1.77 | .876 |
| Social support (F-SOZU) | 182 | **0.62** | **0.44** | **0.87** | **.006** | 167 | 0.65 | 0.41 | 1.03 | .067 |
| Stigma ("I'd be ashamed if I had a mental disorder") | 208 | 0.72 | 0.49 | 1.07 | .102 | 167 | 0.66 | 0.39 | 1.12 | .119 |
| Subjective physical health | 185 | 1.30 | 0.89 | 1.90 | .169 | 167 | 1.08 | 0.66 | 1.77 | .760 |

*Note*. OR: Odds Ratio. [95% CI]: 95% Confidence Interval. OCD: Obsessive Compulsive Disorder. ADHD: Attention Deficit Hyperactivity Disorder. Significant results are printed in bold.

[a] Diagnostic categories that correspond to ICD-10 F3-disorders (Any Bipolar Disorder, Any Depressive Disorder) and F9-disorders (ADHD, Any Disruptive, Impulse-Control or Conduct Disorder) enter as only one category each into multiple regression analyses due to small cell sizes of Any Bipolar Disorder and ADHD; see Table 1). [b] Number of diagnostic categories doesn't enter into multiple regression analyses due to collinearity with diagnostic categories. [c] All scales are standardized. [d] There were no males with Any Eating Disorder (see Table 3).
